# Changes in life history and morphological traits over 8 generations in the brown marmorated stink bug (Hemiptera: Pentatomidae) under mass-rearing conditions

**DOI:** 10.1093/jisesa/ieaf054

**Published:** 2025-05-16

**Authors:** Giacomo Bulgarini, Clara Frasconi Wendt, Manfred Wolf, Angelika Gruber, Leonardo Calabrò, Antonio Pignalosa, Stefanie Fischnaller

**Affiliations:** Laimburg Research Centre, Vadena (BZ), Italy; Laimburg Research Centre, Vadena (BZ), Italy; Laimburg Research Centre, Vadena (BZ), Italy; Laimburg Research Centre, Vadena (BZ), Italy; Laimburg Research Centre, Vadena (BZ), Italy; Laimburg Research Centre, Vadena (BZ), Italy; Laimburg Research Centre, Vadena (BZ), Italy

**Keywords:** Brown marmorated stink bug, mass-rearing, life history traits, fecundity, mortality

## Abstract

Developing and establishing a permanent insect population under mass-rearing conditions is challenging, but it offers the opportunity to collect and compare life history, physiological, morphological, and behavioral traits in real-time and over multiple generations. *Halyomorpha haly*s (Stål, 1855) (Hemiptera: Pentatomidae), a serious agricultural insect pest in northern Italy, was used to establish a permanent mass-rearing protocol under controlled abiotic conditions. The study aimed to evaluate the impact of permanent laboratory rearing on various life history and morphological traits over 8 generations. Development time and developmental success rate of the eggs and nymphal stages, fecundity, mortality rate and body size of the adults were documented. In general, a significant variability was observed in both developmental success rate and developmental time for eggs and juvenile stages, although without an obvious trend. In adults, on the other hand, a common trend in fecundity, number of egg masses and survival was observed. All 3 parameters exhibited a marked decline beginning in the second generation, followed by a significant recovery starting from the seventh generation, indicating potential laboratory adaptation. The body size, on the other hand, showed a slight decrease from the second generation that remained almost constant in subsequent generations. While the results demonstrate the clear success of a continuous *H. halys* mass-rearing, they also show the current challenges and limits of rearing this invasive insect species under laboratory conditions over several generations without the addition of new individuals.

## Introduction

Mass-rearing methods are reliable as they provide a constant number and good quality of individuals throughout the period of the experiment. Additionally, they are less expensive than relying on the private market to buy test animals (Huynh et al. 2021). Furthermore, maintaining a viable, stable, and healthy laboratory population is important for guaranteeing that outcomes of an experiment are attributable to and depend on the treatment. In temperate climates, insect mass-rearing ensures a continuous supply of individuals, allowing experiments to run year-round rather than being confined to spring and summer when most insects are active (Huynh et al. 2021). This approach also guarantees a stable number of eggs, nymphs, and adults even during the cold season, when many insects overwinter and are not available in the field (Taylor et al. 2017). In the context of classical biological control, maintaining significant quantities of host organisms is crucial for producing adequate populations of control agents (Sabbatini-Peverieri et al. 2020). However, mass-rearing can lead to changes in life history traits such as fecundity, mortality, developmental time, longevity, and morphological characteristics (van Bergen et al. 2013, Heydorn et al. 2019). Over several generations, laboratory mass-reared insects may undergo genetic, physiological, morphological and behavioral changes due to the artificial conditions under which they are reared (Miyatake 1998). Specifically, genetic drift, such as inbreeding, founder effect or rearing protocols and artificial selection pressures may cause alterations in insect life history and behavioral and physiological traits (Clercq et al. 1998, Liu et al. 2015, Ueno 2015). For example, Ueno (2015) reported that, in the ectoparasitic wasp *Agrothereutes lanceolatus* (Walker, 1874) (Hymenoptera: Ichneumonidae) reared under laboratory conditions, inbreeding caused a decrease in offspring survival and an increase in the number of male individuals. However, while genetic factors may play a determinant role in such changes, abiotic conditions, such as relative humidity, temperature and nutrient limitation, may have a determinant effect on insect life history and morphological traits too (Deshpande et al. 2015).

To minimize external and internal factors that drive changes in a mass-reared population, it is essential to develop a well-defined protocol that serves as a comprehensive framework. A well-defined protocol refers to a set of standardized procedures designed to achieve specific objectives. This protocol should integrate clear procedures for key tasks, such as environmental control, feeding schedules, and genetic management, to ensure a stable laboratory population and reproducibility across experiments. While these protocols may vary between laboratories, they generally include guidelines on environmental conditions, feeding, and data collection. For example, in insect reproduction studies, the protocol would outline the necessary conditions for monitoring egg-laying and calculating fecundity. In biocontrol programs, the focus would be on rearing healthy organisms for pest control, with procedures to optimize their survival and reproductive success (Sørensen et al. 2012). By establishing a clear, structured protocol, researchers can ensure consistency and reliability, minimizing the influence of uncontrolled factors and achieving reproducible results. Reproductive traits, such as fecundity, frequency of oviposition and mating, and life history traits, namely developmental success rate, longevity and preoviposition period, are some of the most common life history traits used, together with behavioral and physiological traits, such as courtship and hormone production, to evaluate the success of laboratory (mass-) reared insects (Miyatake 1998, Hoffmann and Ross 2018, Momemian et al. 2022).

The brown marmorated stink bug, *Halyomorpha halys* (Stål, 1855) (Hemiptera: Pentatomidae), is an invasive agricultural pest (Leskey et al. 2012, Rice et al. 2014, Leskey and Nielsen 2018). Its original range includes Taiwan, Japan, Korea, and China (Lee et al. 2013, Haye et al. 2015), but nowadays the species has been introduced in several countries throughout the world (Hoebeke and Carter 2003, Wermelinger et al. 2008, Faundez and Rider 2017, Cianferoni et al. 2018, Claerebout et al. 2018). In Europe, the species was first recorded in Switzerland and Liechtenstein in 2004 (Arnold 2009, Haye et al. 2014), while the first individuals were recorded in Italy in 2007 (Cianferoni et al. 2018). *Halyomorpha halys* has 5 nymphal stages and is a polyphagous species, feeding on many different agricultural and ornamental plants (Rice et al. 2014, Lee 2015, Bergmann et al. 2016). Feeding damage by *H. halys* has been reported for several economically important crops and fruits, including soybeans, apples, peaches, and hazelnuts (Rice et al. 2014, Haye et al. 2015). In 2010, apple production losses in the mid-Atlantic region of the USA exceeded $37 million (United States Apple Association 2010). By comparison, in 2019, damage caused by *H. halys* in northern Italy led to an estimated annual economic loss of approximately $658 million in fruit production (CSO Italy 2020). Due to its role as a major pest in many agricultural systems, many laboratories depend on a well-functioning breeding stock of *H. halys* to conduct experiments or produce parasitoids for biological control in the field (Sabbatini-Peverieri et al. 2020, Bittau et al. 2021, Bulgarini et al. 2021). As is the case for many other insect species, different mass-rearing protocol*s* have been proposed for *H. halys* (Medal et al. 2012, Dingha and Jackai 2017, Taylor et al. 2017). Protocols differ in several aspects, but all provide some advantages and challenges for the rearing of this invasive bug species. Initial attempts to develop a method for establishing a laboratory population of *H*. *halys* with more than 1 generation came from Medal et al. (2012), who succeeded in producing first generation (F1) eggs. The size of insect cages, food supply, abiotic conditions in the rearing chambers and the number of individuals per cage are just some of the factors that vary between different protocols and may influence the success of mass rearing of this invasive insect. With regard to food source, many diets have been tested for *H. halys*, and the first efforts showed that a mixed diet based on carrots, peanuts, and soybeans could substantially improve the success of rearing compared to a single food source (Funayama 2006, Acebes-Doria et al. 2016). Recently, Dingha and Jackai (2017) tested different food sources, namely fresh vegetables, fruit and shrub leaves, and their effects on specific life history traits of *H. halys*. They showed that developmental success rate, time, and survival increased when *H. halys* was fed with around 15 different food sources. However, besides the fact that providing such a large variety of food sources over time may come with some costs, the sizes of the commonly used bug dorms (Rosen et al. 2016) may represent another limiting factor.

Most studies have reared the invasive stink bug until F1 and are limited to up to 3 mo or a few generations (Medal et al. 2012, Dingha and Jackai 2017), and to our knowledge a long-term experiment performed over several generations without the repeated addition of individuals from wild populations has yet to be conducted. A permanent mass-rearing system is a stable, self-sustaining colony designed to produce multiple generations of individuals under controlled conditions, ensuring a continuous supply for long-term research without the need to introduce wild-caught specimens. Here, we adapted protocols proposed in past studies (Medal et al. 2012, Acebes-Doria et al. 2016, Rosen et al. 2016, Dingha and Jackai 2017, Taylor et al. 2017, Sabbatini-Peverieri et al. 2020) and documented several life history and morphological traits to establish a permanent *H. halys* mass-rearing system, capable of supporting long-term research. Our objective was to evaluate the success of its mass-rearing, while advancing a protocol for a permanent laboratory population of *H. halys*. To do so, changes in life history such as fecundity, developmental time, and developmental success rate, and mortality, as well as morphological traits, specifically body size, were documented across multiple generations. The number of eggs per egg mass, number of egg masses per female (fecundity), developmental time in days, and degree days (DD) from egg to adult, as well as developmental success rate and survival were recorded.

## Materials and Methods

### *H. halys* Rearing

With a view to establishing a permanent laboratory population of *H. halys*, adult individuals were actively sampled in the field from September to October 2020 twice a week, from 8 AM to 2 PM, by using a white polyethylene fabric beating sheet (W1 × L1 m) or by visual inspection of building walls. The sampling of many adults was simplified by the aggregation behavior *H. halys* demonstrates when entering into diapause (Leskey et al. 2012), this behavior allowed the sampling of many individuals at once. To ensure high genetic diversity, samples were collected from different areas of South Tyrol (Italy), encompassing agricultural, urban and peri-urban sites. Additionally, an official invitation was issued via press release, encouraging private individuals to collect samples and bring them to the laboratory. The number of individuals collected from each area was not specifically documented, though the range varied from a minimum of 29 to a maximum of 149 individuals. The collected individuals, which represented the parental generation (F0), were transferred into insect cages (BugDorm; BugDorm-1 insect rearing cage, dimensions: W30 × L30 × H30 cm; MegaView Science Co., Ltd., Taichung, Taiwan) in cohorts consisting of 60 individuals, mixing individuals from different sampling date and locations. Cohorts were provided with 40 g of *Daucus carota* (Linnaeus, 1753) (carrot), 90 g of *Phaseolus vulgaris* (Linnaeus, 1758) (green bean), 7 g of *Helianthus annuus* (Linnaeus, 1753) (sunflower) seeds and 2 caps from 50 ml centrifuge tubes filled with cotton soaked in tap water as nourishment. Rearing cages were placed into a climatic chamber (Criocabin; Genesis System SP.70, dimensions: W2100 × L3600 × H2100 mm; Criocabin S.p.A., Veneto, Italy) at 19 °C under short-day conditions (light:dark cycle 10:14 (L:D)) for about 2 to 3 wk. After this “prediapause” setting, individuals experienced a 143-d diapause phase (9 °C, 8:16 (L:D)) in the same climatic chamber without food supplies, but with paper towels (W25 × L25 cm) and brown, single-wave carboard (14.5 × 15.5 cm, 3 mm thick) to use as shelter. Cardboard pieces were folded or rolled up as much as possible and cages were filled with paper towels and cardboard to maximize shelter availability. By the end of this period, mortality rates were documented, and some of the survived adults (F0) were used to start permanent rearing. Individuals from different diapause insect cages were sexed according to Rice et al. (2014) and mixed to form new insect cages for the mass-rearing. Each F0 cohort consisted of 25 females and 25 males. The rearing population was kept in climatic chambers, with conditions set at 25 °C, 65% relative humidity (RH) and 16:8 (L:D). RH was maintained through a computer-controlled water sprinkler, which sprayed aerosol drops every 10 min for 6 s whenever the humidity dropped below 60%. The water spray was delivered via the Air Jet Fogger (Model 23413.10 CL S.S.CO, TeeJet, Glendale Heights, Illinois, USA). To ensure optimal experimental conditions, we used deionized water with a conductivity of 6 µS/cm, minimizing ionic content and reducing the risk of contamination or interference with the setup. Adults were fed with green beans and carrots that had been washed with running water, sunflower seeds, and tap water-soaked cotton in the same quantities as detailed previously, partially following Taylor et al. (2017) and Sabbatini-Peverieri et al. (2020). A crumpled paper towel and a folded cardboard piece were provided as shelter. Two pieces of 100% cotton-gauze net (W20 × L20 cm) were added, as previous experiments indicated them as preferred oviposition sites. The following procedures were carried out for each generation of *H. halys* rearing populations, with each generation being strictly isolated from one another.

As soon as adults started laying eggs, each egg mass was removed from the cage, the number of eggs counted under a stereomicroscope and placed individually in a glass Petri dish (Glass Petri dish, soda-lime 80 × 15 mm; Normax, Marinha Grande, Portugal). Egg masses were reared in the climatic chamber under the same temperature, RH and photoperiod conditions of the adult cohorts, with a piece of green bean provided until the eggs hatched and developed into second instar nymphs (N2). A minimum of 90 egg masses per generation were maintained to continue the rearing program, following the previously described protocol. Due to space constraints, not all egg masses were retained for rearing. Egg masses deposited after the capacity of the climatic chamber was reached were recorded, but the number of eggs was not quantified, and instead these were allocated for alternative experimental purposes. Following Dingha and Jackai (2017), only N2 individuals were transferred to the nymphal rearing cages to minimize mortality, as first instar nymphs (N1) tend to aggregate and are difficult to handle. Fresh molted N2 individuals were removed from Petri dishes of the same generation, mixed and placed into rearing cages in groups of 100 per cage. They were maintained under these conditions until they molted into adults. The nymphs were fed a diet identical to that of the adults, with the addition of one *Actinidia chinensis* (Planchon, 1847) (green kiwi). The use of standardized food sources eventually allowed researchers to compare the life history and morphological traits of *H. halys* over generations. Freshly molted adults were then transferred into new adult cages for reproduction. Eggs, N2 to fifth instar nymphs (N5) and adults were maintained separately to avoid cannibalistic oophagy, which has been observed in previous mass-rearing experiments (Iverson et al. 2016).

Twice a week (Monday and Thursday) food and water were replaced, and the cages for both nymphs and adults were cleaned with ethanol diluted to 70%. Maintenance started at 8 AM and lasted from 2 to 8 h, depending on the number of cages and Petri dishes to be serviced. Two operators were responsible for food replacement and cage cleaning. At each maintenance session, defined as a scheduled cleaning and feeding event, the number of dead individuals in adult cohorts and freshly produced egg masses were recorded. Each adult cage for each generation hosted 25 pairs of adults, and no individuals were replaced as they died. A cage was dismantled when (i) all the individuals were dead, (ii) all the females were dead or (iii) females did not lay eggs for 3 consecutive maintenance sessions. The number of replicates for each life stage and generation is shown in Table 1.

**Table 1.** Number of egg masses, nymphal cohorts, and adult cohorts for each generation.

Generation	Egg masses (N°)	Nymphal cohorts (N°)	Adult cohorts (N°)
F0	×	×	5
F1	145	32	15
F2	266	20	14
F3	129	21	14
F4	191	24	18
F5	351	17	9
F6	125	23	9
F7	95	12	12
F8	175	11	11

### Measurement of Life History and Morphological Traits of *H. halys*

#### Life History Traits

The separation of the nymphs and adult stages into different rearing cages and the separate collection of eggs in Petri dishes allowed the recording of specific parameters for each developmental stage. These parameters were subsequently used to calculate life history traits for each generation. Changes in various fitness-related parameters, such as fecundity, mortality, development and dimension were recorded. These parameters are some of the life history traits most used for assessing changes over time in mass-rearing (Hoffmann and Ross 2018, Li et al. 2021, Momemian et al. 2022). The specific parameters recorded, and their corresponding life history traits are listed in Table 2.

**Table 2.** Life history traits collected for each developmental life stage. Developmental life stage, biweekly parameter recorded, and the corresponding life history trait are listed below. Parameters were recorded for each insect cage separately. The times regarding the developmental, the appearance of first and last adults and the preoviposition period were expressed in DD.

Development life stage	Parameter recorded	Correspondence
1.Egg mass—second instar	Developmental time (DD)	Development
	N° nymphs developed	
2. second instar—adult	Developmental time (DD) Appearance first adult (DD)	
	Appearance last adult (DD)	
	N° adults developed	
	N° females and males developed	
3.Adult	Body length (mm)	
	Pre-oviposition period (DD)	
	N° egg masses	Fecundity
	N° egg masses per female	
	N° deaths during time	Mortality

#### Morphological Traits (Body Size)

Morphologicto assess changes in adult body size over time, dead adults from F0 to the seventh generation (F7) were collected, labeled (insect cage where they were collected, sex and date of collection) and kept in a freezer at –20 °C for further investigation. Frozen individuals were placed separately on plasticized graph paper under a stereomicroscope (Leica; Leica M205 C, zoom 0.78×, brightfield illumination; Leica Microsystems GmbH, Wetzlar, Germany), photographed (Leica; Leica IC80 HD; Leica Microsystems GmbH, Wetzlar, Germany) and labeled (generation and sex). The photos were not altered in any way. For the measurements, the ImageJ program (version 1.53r, 2022) was used. For each measurement session, a scale was established by measuring the number of pixels between 2 points on graph paper with a known distance of 1 mm. Using this scale, the distance from the apex of the head to the end of the abdomen was measured by drawing a line with the mouse. The software then provided the measurement in millimeters, calculated by counting the pixels in the line based on the initial calibration.

### Data Analysis

To assess changes in life history traits, both the total number of days and the DD were considered. The total number of days refers to the number of days needed for a certain process/development, eg the number of days needed for the development from egg to N2. As temperature plays an important role in the growth and development of insects (Ratte 1984, Wolda 1988), a more accurate way of expressing insect development is to calculate the number of DD (Dixon et al. 2009, Damos and Savopoulou-Soultani 2012). The DD considers the sum of the potential number of hours for the optimal development in a day within a certain range of temperature. Various ranges of critical temperature for successful development of *H. halys* can be found in the literature (Baek et al. 2017, Govindan and Hutchison 2020, Mermer et al. 2023). To calculate daily DD we used a critical temperature threshold (*T*_0_) of 13 °C (Nielsen et al. 2008, Haye et al. 2014, Musolin et al. 2019). We used the formula:


$$\begin{array}{l} DD=\frac{\overset{n}{\underset{i=1}{\sum }}(T_{base}-T_{0})}{24} \end{array}$$

where *n* is the number of days considered and *T*_*base*_ is the hourly temperature maintained in the climatic chamber.

The development time, expressed in DD, from egg to adult for each generation was calculated in 2 steps: first from egg to N2 and then from N2 to adult for each cohort, also considering the emergence of the first and last adult. Developmental success rates were quantified as the ratio of viable N2 to the total number of eggs and the ratio of viable adults to the total number of N2. These measurements were recorded for each egg mass and each juvenile insect cage, respectively. The sex ratio of developed adults was calculated as the percentage difference between the numbers of developed adult males and females, relative to the initial number of N2 individuals. Fecundity was measured for each cage as the sum of the ratios of the number of egg masses laid to the number of surviving females in each maintenance session (twice a week).

To assess whether different recorded parameters could be influenced by generation, a one-way ANOVA was performed with generation as the independent variable. For the parameters related to egg masses (development time in DD and developmental success rate), individual egg masses were considered as replicates. For the development from N2 to adult (general development time, the appearance of the first and last adult, all measured in DD, the developmental success rate, and the number of developed males and females), each parameter was calculated for each cage, which was thus considered as a replicate. The same approach was applied to the parameters related to adults (the preoviposition period in DD, the number of egg masses, and fecundity). For individual measures, each specimen was treated as a replicate. Where significant differences arose, the posthoc Tukey’s (HSD) test was used. The “survival” package was used to assess difference in adult mortality across generations over time, measured in weeks. Specifically, survival times for each generation were calculated, and pairwise comparisons of survival curves were performed using the “survdiff” function, which conducts the log-rank test to evaluate the significance of differences between curves. When significant differences were detected, the “survminer” package was employed to visualize the survival curves and highlight these significant differences between generations. All statistical analyses were performed using Rstudio (version 2023.6.0.0, RStudio Team, 2023). Although not used for statistical analyses, the average adult survival per generation was also estimated by calculating the difference between the start and end date of each cage. Cages were treated as replicates for each generation, and survival was expressed in both days and DD.

## Results

Eight consecutive generations of *H. halys* were reared under controlled laboratory conditions, taking 1,280 h for each operator in 20 mo. More than 1,600 egg masses were collected, over 160 nymph cages and more than 100 adult cages were set up. For the rearing of the various generations, between 32% (F7) and 95% (second generation; F2) of the produced egg masses were utilized. Table 1 shows the numbers of egg masses, nymphal cohorts, and adult cohorts along with respective generations. Different generations showed a different mean number of egg masses as well as different rates of developmental success (from N2 to adult), fecundity, mortality, and morphological traits.

### Egg to N2

**Fig. 1.**
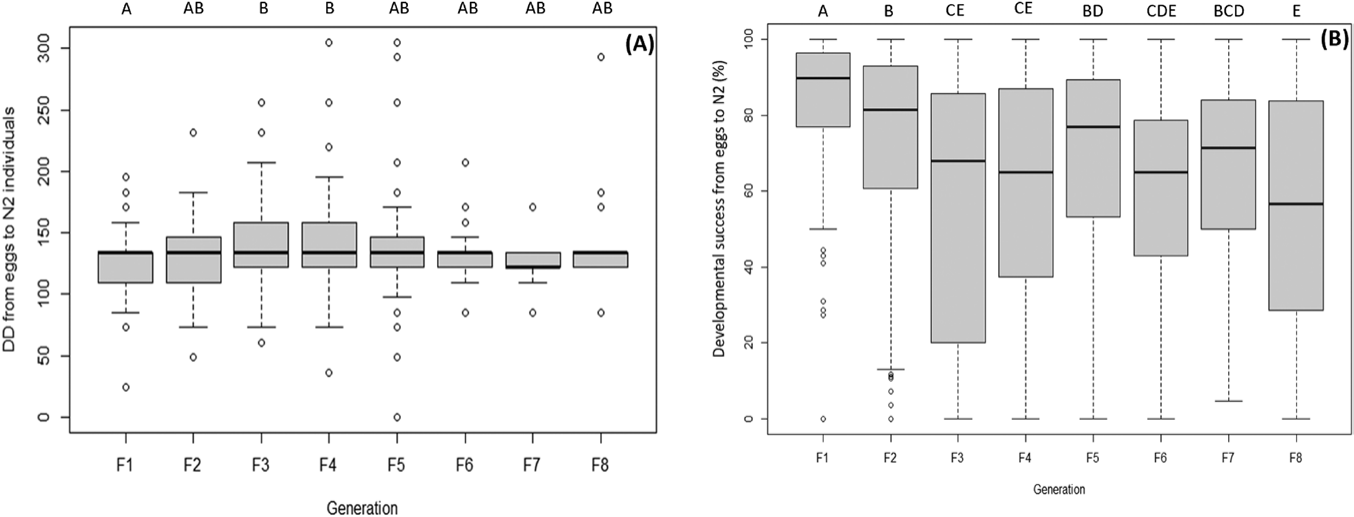
A) DD (±SE) necessary to develop from eggs to N2 for each generation. B) Developmental success rate (±SE) from eggs to N2 for each generation. Different letters on top of bars indicate statistically significant differences between the generations (Tukey’s HSD: *P* < 0.05).

For the egg stage, the results showed a significant difference between generations for the 2 parameters considered: the developmental time (DD) (*F* = 3.2; df = 7, 1426; *P* = 0.002) and the developmental success rate from eggs to N2 (*F* = 17.7; df = 7, 1469; *P* < 0.001). The third (F3) and fourth generation (F4) egg masses needed more DD to develop to N2, while F1 needed less. From F3, it took more DD to develop and then remained constant in subsequent generations (Fig. 1; Table 3). F1 had a higher developmental success rate, with a mean of 83%, while F3, F4 and the eighth generation (F8) had the lowest with mean values that did not reach 60%. It looks like the developmental success rate varied unevenly between generations, with a regular decline until F3 and then increasing slightly in the fifth (F5) and declining again until F8 (Fig. 1; Table 3).

**Table 3.** Summary of key egg mass development parameters for *Halyomorpha halys* across 8 generations

Generation	Eggs	Mean eggs per egg mass	Calculated estimate total eggs	Percentage of egg masses used	N° N2	Developmental success rate E-N2 (%)	Developmental time E-N2 (d)	Developmental time E-N2 (DD)
F1	3,717	26	7,511	49%	3,147	83.4 ± 1.7	10.4 ± 0.2	128.0 ± 2.0
F2	6,612	25	19,140	35%	4,772	71.5 ± 1.7	10.7 ± 0.1	131.1 ± 1.6
F3	3,091	24	3,259	95%	1,761	55.9 ± 3.2	11.5 ± 0.2	139.9 ± 2.9
F4	4,553	24	5,101	89%	2,735	58.7 ± 2.4	11.3 ± 0.2	138.2 ± 2.5
F5	8,273	24	11,361	73%	5,688	67.7 ± 1.6	11.1 ± 0.1	134.9 ± 1.6
F6	2,927	23	3,536	83%	1,724	59.5 ± 2.5	10.9 ± 0.1	132.4 ± 1.7
F7	2,132	22	4,084	52%	1,443	66.7 ± 2.5	10.7 ± 0.2	130.7 ± 2.1
F8	4,301	25	13,493	32%	2,368	54.5 ± 2.3	11.0 ± 0.1	134.3 ± 1.7

### N2 to Adult Stage

For the nymphs, significant differences were observed in the accumulated DD needed to develop all N2 into adults, the DD necessary for the development of the last adult, and the rate of developmental success rate (*F* = 9.3; df = 7, 162; *P* < 0.001; *F* = 4.8; df = 7, 162; *P* < 0.001 and *F* = 10; df = 7,52; *P* < 0.001, respectively).

**Fig. 2.**
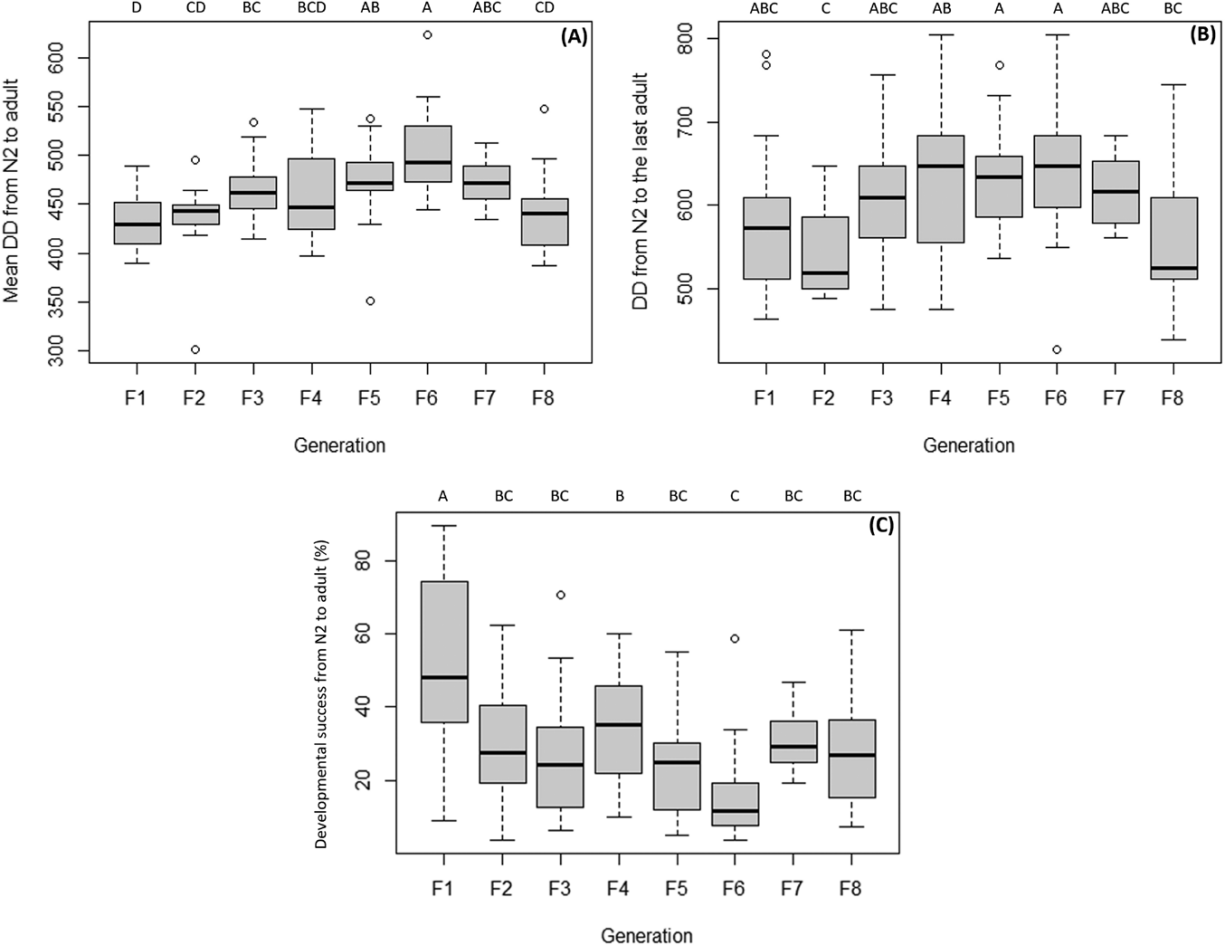
A) Mean of the overall DD (±SE) necessary to develop from (N2) to adult for each generation. B) DD (±SE) necessary to develop all the N2 individuals into adults for each generation. C) Developmental success (±SE) from N2 to adult for each generation. Different letters on top of the bars indicate statistically significant differences between the generations (Tukey’s HSD: *P* < 0.05).

In terms of DD, F1 needed fewer heat units to develop from N2 to adult, with a mean of 431 DD, while F5 and the sixth generation (F6) required more time with an average of 475 and 502 DD, respectively. Regarding the DD necessary to develop all the N2 into adults, F2 needed fewer heat units, with a mean of 538 DD, while F5 and F6 required more developmental time, with an average of 630 and 639 DD, respectively. The DD for the development from N2 to adult varies between generations, with an increase from F3, then remains constant and decreases again in F8 (Fig. 2; Table 4).

**Table 4.** Summary of key juvenile development parameters for *Halyomorpha halys* across 8 generations

Generation	N2	N° Adults	M	F	Developmental success rate N2-A (%)	Developmental time (d)	Developmental time (DD)	Developmental time (d) 1° Adult	Developmental time (DD) 1° Adult	Developmental time (d) last Adult	Developmental time (DD) last Adult
F1	2.212	1,125	586	539	51.5 ± 4.7	35.34 ± 0.4	431.1 ± 5.3	30.1 ± 0.8	367.9 ± 9.8	48.2 ± 1.5	588.5 ± 18.1
F2	2.444	697	395	302	29.7 ± 3.6	35.8 ± 0.6	436.7 ± 8.1	30.2 ± 1.0	369.0 ± 12.1	44.1 ± 0.9	538.6 ± 10.5
F3	2.224	538	284	254	25.9 ± 3.6	38.17 ± 0.5	465.6 ± 6.9	31.8 ± 0.7	388.0 ± 9.0	49.9 ± 1.3	609.4 ± 15.4
F4	2.574	847	404	443	33.3 ± 3.1	37.78 ± 0.7	460.8 ± 9.6	30.7 ± 0.8	375.0 ± 10.1	51.6 ± 1.5	630.1 ± 18.2
F5	3.148	733	383	350	23.8 ± 2.7	39 ± 0.6	475.8 ± 7.5	31.1 ± 0.7	379.6 ± 8.2	51.7 ± 1.0	630.9 ± 12.0
F6	2.839	468	236	222	16.0 ± 2.6	41.17 ± 0.7	502.3 ± 8.9	33.0 ± 0.8	402.6 ± 9.4	52.3 ± 1.5	639.1 ± 18.2
F7	1.480	447	239	208	30.6 ± 2.4	38.69 ± 0.5	471.9 ± 6.9	30.0 ± 0.5	366.0 ± 6.5	50.5 ± 1.0	617.1 ± 12.4
F8	2.583	709	367	342	26.9 ± 3.1	36.01 ± 0.7	439.5 ± 8.4	30.1 ± 0.7	367.7 ± 8.1	46.0 ± 1.6	562.3 ± 19.0

F1 had the highest developmental success rate from N2 to the adult stage with a mean of 51%, while F6 had the lowest rate with a mean of 16%. The developmental success rate varies with the progression of the generations, with a sharp decline starting from F2, then remaining constant until F8 with a decline in F6 (Fig. 2; Table 4).

### Adults

For the adults, all the parameters considered showed a significant difference between the generations. The ANOVA and survival test results are reported in Table 5.

**Table 5.** Statistical results for each parameter, with ANOVA (df, *F*, and *P*-values) and survival test (df, *χ*², and *P*-values)

Parameters	df	*F*	*P*
Egg masses	8, 94	10.8	<0.001
Fecundity	8, 94	12.8	<0.001
Dimensions	7, 2731	39.7	<0.001
DD before first egg mass	8, 94	3.5	0.001
		***χ*** ^***2***^	
Survival	8	2376	<0.001

**Fig. 3.**
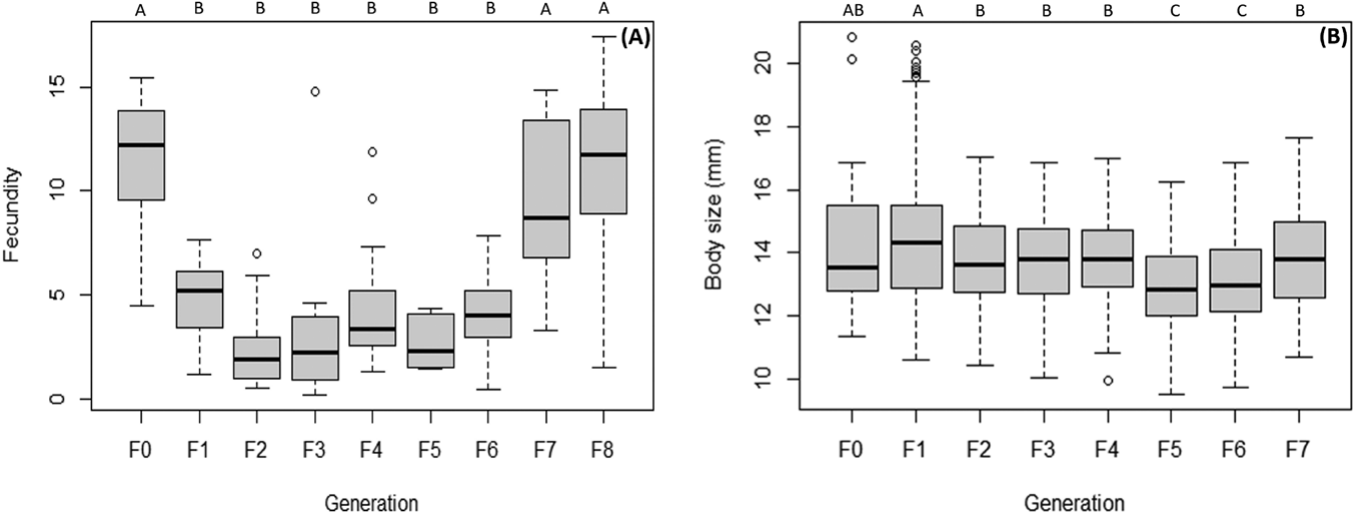
A) Fecundity (Number of egg masses/Number of vital females) in each generation. B) Body length of adults (from the apex of the head to the end of the abdomen) in each generation. Different letters on top of the bars indicate statistically significant differences between the generations (Tukey’s HSD: *P* < 0.05).

Adults of F1, F7, and F8 laid the most egg masses: 770, 549, and 632 respectively, while F2 laid the fewest, with 136 egg masses. The other generations did not differ significantly from F2. While F0 and F1 showed no significant differences, a decline in egg masses depositions started with F2. This decline was followed by an increase starting from F7 onward (Table 6). A similar trend is observed in fecundity, being linked to the number of egg masses laid per female. F0 and F8 exhibit the highest average fecundity with 11 egg masses per female, followed by F7 (mean = 9 egg masses per female). F2 and F5 had the lowest fecundity with an average of 2 egg masses per female. Thus, the calculated fecundity decreased starting from F1, remained constant across subsequent generations until an increase beginning at F7, with no significant difference between F0, F7, and F8 (Fig. 3; Table 6).

**Table 6.** Summary of key reproductive, morphological, and survival parameters for adult *Halyomorpha halys* across 8 generations of mass rearing

Generation	Egg masses	Fecundity	Body length F (mm)	Body length M (mm)	Preoviposition period (d)	Preoviposition period (DD)	Survival (d)	Survival (DD)
F0	293	11.1 ± 1.9	15.9 ± 0.7	13.4 ± 0.2	17.0 ± 0.8	207.4 ± 9.5	94.0 ± 7.0	1,146.8 ± 85.7
F1	770	4.7 ± 0.5	15.6 ± 0.1	13.1 ± 0.1	10.1 ± 0.6	122.8 ± 7.8	63.6 ± 3.1	775.9 ± 37.9
F2	136	2.5 ± 0.5	14.8 ± 0.1	12.8 ± 0.0	12.5 ± 1.3	152.5 ± 15.8	33.8 ± 2.6	412.2 ± 31.1
F3	214	3.1 ± 1.0	14.6 ± 0.1	12.8 ± 0.1	9.9 ± 1.0	120.3 ± 12.1	37.6 ± 3.4	459.2 ± 41.1
F4	482	4.4 ± 0.7	14.5 ± 0.1	12.8 ± 0.1	13.1 ± 1.1	160.0 ± 13.1	45.5 ± 2.2	555.1 ± 27.0
F5	151	2.7 ± 0.4	13.6 ± 0.1	12.0 ± 0.1	11.6 ± 1.3	141.0 ± 16.0	44.1 ± 2.3	538.2 ± 27.8
F6	182	4.2 ± 0.7	14.0 ± 0.1	12.1 ± 0.1	10.7 ± 0.9	130.1 ± 11.5	41.8 ± 4.7	509.7 ± 56.9
F7	549	9.5 ± 1.4	14.9 ± 0.1	12.7 ± 0.1	9.6 ± 1.2	117.4 ± 15.1	63.4 ± 5.1	773.2 ± 61.6
F8	632	11.0 ± 1.4	×	×	9.1 ± 0.7	110.9 ± 8.8	80.2 ± 5.6	978.2 ± 68.8

Regarding body size, there was a significant distinction between the generations, with F1 exhibiting the largest dimensions (on average 15.56 mm for females and 13.12 mm for males), which was not significantly different from F0. The decrease in body size observed in F2 remained relatively constant until F4, followed by a further decrease, with F5 and F6 having the smallest dimensions (13.64 mm for females and 12 mm for males and 14 mm for females and 12.11 mm for males, respectively). F7 returned to dimensions that were not significantly different from those recorded for F2, F3, and F4 (Fig. 3; Table 6).

**Fig. 4.**
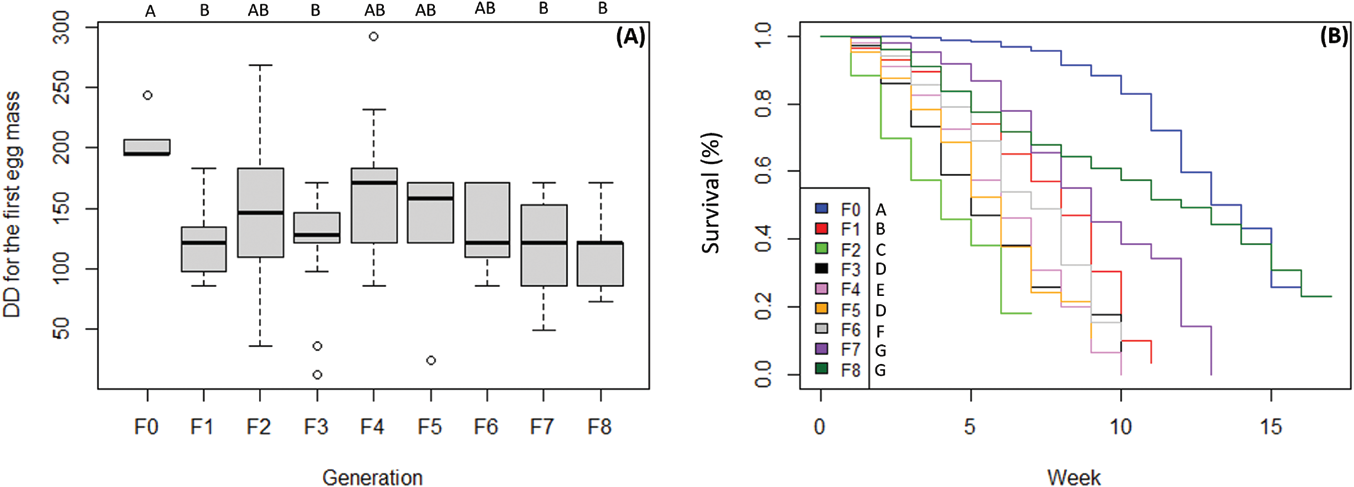
A) DD required for first oviposition in each generation. Different letters on top of the bar indicate statistically significant differences between the generations (Tukey’s HSD: *P* < 0.05). B) Survival over time (in weeks) in each generation. Different letters next to the legend indicate statistically significant differences between the generations (Survminer package *P* < 0.05).

The preoviposition period, calculated from the start of each adult cohort to first deposition of the first egg mass, did not change significantly between generations, except for F0, which required more DD (on average 207.4 DD) (Fig. 4; Table 6).

Survival varied across generations. F0 had the longest lifespan, with a mean of 94 d, reaching the 16th week with 26% of individuals still alive. In contrast, F2 had the shortest lifespan, with a mean of 34 d, reaching the seventh week with 18% of individuals still alive. Significant differences in survival were observed between all generations, except between F3 and F5, which reached the 10th and the 9th week with 7% and 11% of individuals still alive and had a mean lifespan of 38 and 44 d, respectively. F7 and F8 also showed no significant difference from each other. F7 had a mean lifespan of 63 d, with all individuals dead by the 13th week, while F8 had a mean lifespan of 80 d, reaching the 17th week with 23% of individuals still alive. Although F8 persisted 1 wk longer than F0, it exhibited lower overall survival due to an early and sharp decline. Specifically, F8 experienced a sharp drop in survival during the initial weeks, whereas F0 maintained high survivals for a longer period. In F0, survival remained above 90% until the eighth week and declined to 50% by the 13th week. In contrast, F8 maintained survival above 90% only until the third week and reached 50% by the 12th week. This distinction is evident in the survival curves, which show that F0 and F8 follow distinct trajectories (Fig. 4). Survival was highest in F0, decreased in F1, and further declined in F2. From F3 onwards, survival increased with some variation, until F7 and F8, which had greater survival than F1 but lower than F0 (Fig. 4; Table 6).

## Discussion

We successfully reared *H. halys* for 8 generations and over 20 mo under controlled laboratory conditions without introducing new individuals from external sources. Furthermore, we provided a detailed protocol for mass-rearing of *H. halys*. Previous studies have typically examined fewer generations over shorter time periods (Medal et al. 2012, Acebes-Doria et al. 2016, Rosen et al. 2016, Dingha and Jackai 2017, Taylor et al. 2017). To our knowledge this is the first attempt to establish a stable, autonomous mass-rearing population of *H. halys* without the addition of wild-caught individuals throughout the entire duration of the experiment. Adapting the existing protocols allowed us to overcome several problems linked to the practical aspect of insect mass-rearing too. For example, physically separating the different developmental stages from egg to N2 in Petri dishes, N2 to adult in nymphal cages and adults in adult only cages enable detailed tracking of all life history traits and minimized cannibalistic oophagy (Iverson et al. 2016). The life history and morphological traits recorded over several generations allowed us to assess changes in developmental time and success, fecundity, mortality, and morphology over time and to compare the fitness between generations.

With respect to at least one of the parameters evaluated, generational differences were observed in all developmental stages (eggs, juvenile, and adults). Considering the egg masses, the DD required for development from hatching to N2 varied across generations, showing an increase starting from F3 and then stabilizing in subsequent generations. The developmental success rate, on the other hand, exhibited greater variability with F1 and F2 having the highest developmental success rate (means of 83% and 71%, respectively), while F8 showed the lowest developmental success rate (mean of 54%). F3 and F4 did not differ significantly from F8 in terms of developmental success rate, whereas the other generations displayed intermediate values.

The DD required for the development from N2 to the first freshly molted adult did not vary significantly between generations. In contrast, the DD required for the complete development of all individuals and for the last freshly molted adult showed variations between generations. This difference among the 3 categories may be attributed to the fact that the development time from N2 to the first freshly molted adult is based on the progression of a single individual. In contrast, measures for complete development and for the last freshly molted adult depend on the molting of all individuals within a cohort, which extends the overall developmental duration. Additionally, individual variations in developmental success rates, even among siblings, may contribute to the observed trend. These differences could explain why the DD required for the emergence of the last adult and the DD needed for overall development from N2 to the adult stage show similar trends across generations. An increase is observed from F3 to F6, followed by a decline in the last 2 generations, until they equal those of F1. In addition, the 2 categories are closely linked, since to obtain the last adult, all the other individuals had to develop first. The greater variability observed in required DD from hatching to N2, may depend on the fact that individuals must go through 3 additional juvenile stages before reaching the imaginal stage. This requires multiple molts and a greater accumulation of resources, which individuals may not always be able to obtain. In contrast development from hatching to N2 involves only a single molt. Many individuals died during the molting processes, particularly between N5 and the adult stage, leading to a lower proportion of individuals reaching adulthood, which is likely due to insufficient resource accumulation for completion of metamorphosis, despite the availability of food and water ad libitum. Consequently, the developmental success rate from N2 to the adult stage, ranging from 16% to 51%, is lower than that of individuals reaching the N2 stage, which ranged from 54% to 83%. The variability in developmental success rates was lowest in F1 (which had the highest success rate), and in F6 (which had the lowest), while other generations exhibited success rates in an intermediate range between F1 and F6, without significant differences among them. Additionally, there was no significant difference in the number of developed males and females between generations. Thus, the sex ratio appears to be consistent and not influenced by generations, at least up to F8.

There were no significant differences in the duration of the preoviposition period between generations, except for F0, which required a higher number of DD, and therefore a longer time to produce first egg masses of the filial generation. Prior to standard conditions (25 °C, 16:8 L:D), F0 adults experienced short-day conditions for several weeks to simulate overwintering and maintain reproductive diapause status. This transition from nonreproductive to reproductive status may have necessitated additional time for F0 adults to achieve reproductive maturity (Nielsen and Hamilton 2009, Saulich and Musolin 2012, Musolin et al. 2019).

In permanent mass rearing, physical and morphological traits are among those that may exhibit variability between generations. They generally occur more slowly than the other parameters and can also be influenced by factors such as overcrowding in the rearing environment (Hoffmann and Ross 2018, Mahavidanage et al. 2023). This assumption was confirmed by the comparison of adult body size measurement. A decline in body size was observed as early as F2, which remained constant until F5, where a further decline occurred, followed by an increase in F7. However, the body size of F8 remains smaller than that of F1. Our study demonstrates that size varies between generations, though the variation is not pronounced. Except for F5 and F6, the other generations do not differ significantly from F0. These findings align with previous studies, reporting gradual changes in morphological and physiological traits over time (Hoffmann and Ross 2018). To achieve more conclusive information, it would be necessary to perpetuate rearing across additional generations. In each generation, females were consistently larger than males on average, with a maximum size of 15.9 mm in F0 and a minimum of 13.6 mm in F5. Males, in comparison, had a maximum size of 13.4 mm in F0 and a minimum of 12 mm in F5. Additionally, size variability was less pronounced in males than in females, probably because the generally larger size of females results in a broader range of measurements.

A similar trend was observed across fecundity, survival, and number of produced egg masses. F0 exhibited the highest fecundity, the highest number of egg masses produced and the longest lifespan, characterized by a lower mortality over time. F1 showed a significant decrease in all parameters except for the number of egg masses laid. F2 showed the lowest fecundity, producing fewer egg masses and having the shortest lifespan, with a higher mortality rate over time. For all 3 variables, the trend reversed in F7 where fecundity, survival, and number of egg masses increased. F7 was comparable to F0 regarding fecundity and number of egg masses, while survival improved and approached that of F0, exceeding even F1, except for the number of egg masses. F8 did not differ significantly from F7, although it showed differences from F0 in terms of survival. A decline in these variables was anticipated, due to the effect of inbreeding, genetic drift and bottleneck events, the main driving forces of performance declines in mass and continuous rearing, especially when the addition of external individuals is not incorporated (Powell and Evans 2017, Hoffmann and Ross 2018, Ghaemmaghami et al. 2021, Ivey et al. 2023). Interestingly, we observed a performance improvement in F7 and F8. The fact that these later generations exhibited values not significantly different from those of F0 could indicate an adaptation to laboratory conditions. Such adaptation has been observed in several insect species, even after only a few generations (Pratissoli et al. 2004, Hoffmann and Ross 2018, Ivey et al. 2023), and may be driven by unintentional artificial selection. Abiotic factors such as constant temperature and humidity, could have selected for individuals best suited to these conditions. This assumption is supported by the initially high mortality, low fecundity, and low number of egg masses observed in F2 adults, which gradually improved in subsequent generations, reaching more stable levels by F7. The relatively rapid adaptation to laboratory conditions after a fairly low number of generations may have been facilitated by a high genetic diversity of F0 individuals. In fact, an analysis of local populations of *H. halys* revealed a high number of haplotypes present in South Tyrol (Schuler et al. 2021). MtDNA haplotypes may be associated with various genotypic and phenotypic traits (Li et al. 2023), although their dynamics and impacts remain poorly understood and are subject to ongoing debate (Ghiselli and Milani 2020). The presumable high genetic diversity of our starting population (F0), which was collected from various localities across South Tyrol, may have helped overcome bottleneck and facilitated adaptation to laboratory conditions.

An additional factor that may have contributed to adaptive processes in the laboratory setting is the dietary regimen. The quality and diversity of the diet can significantly impact life history and morphological traits and the observed changes over time in our case, may be due to the limited food provided in this study. While we provided individuals with 4 to 5 food sources, depending on the instar stage, in accordance with Nielsen et al. (2008), other studies reported a decrease in development time and an increase in survival when *H. halys* was offered up to 15 different food sources (Dingha and Jackai 2017). For instance, there was a positive correlation between the use of peach foliage and fruiting structure and both survival and developmental success rates of *H. halys*, particularly for nymphal development (Acebes-Doria et al. 2016). Furthermore, food source from the field may offer superior nutritional contents. Taylor et al. (2017) showed that *H. halys* individuals collected from the field and subsequently subjected to diapause exhibited higher survival compared to those maintained in rearing chambers and exclusively fed a diet comprising green beans and raw sunflower seeds before being transferred to diapause condition. This discrepancy underscores the potential impact of foraging in the natural habitat on nutritional quality and survival outcomes (Taylor et al. 2017). Additionally, a relationship between body size and food sources was observed, with individuals of *H. halys* feeding on carrots, raw peanuts, and dry soybeans seeds being larger than individuals with a raw peanut and dry soybeans only diet (Funayama 2006). Dietary constituents can modulate the composition of the microbiome, thereby exerting an influence on the survival capabilities and phenotypic expression of individuals (Acebes-Doria et al. 2016, Luo et al. 2021, Ørsted et al. 2022). The consistent diet may have influenced and/or selected a specific microbiome conducive for survival in the rearing environment, thereby effecting fecundity and mortality. To confirm this hypothesis, the analyzing of the microbiome, such as denaturing gradient gel electrophoresis (DGGE), fluorescent in situ hybridization (FISH) and high-throughput sequencing (HTS) techniques (Shi et al. 2010, Cock et al. 2019), across generations and comparing it to the composition of wild-caught populations would be necessary. However, this specific analysis was beyond the scope of our study.

Factors other than genetic variability and food sources may contribute to the high variability observed in some life history traits. Infections or pathogens play an important role in both mass-rearing systems and wild populations, as they can influence the developmental success rate and survival of infected individuals (Bertola and Mutinelli 2021). For example, in the USA, *Nosema maddoxi* (Becnel, Solter, Hajek, Huang, Sanscrainte & Estep, 2017) (Microsporidia: Nosematidae) was found to be a widespread pathogen in the *H. halys* population, causing substantial nymphal mortality, a deterioration in the lifespan, and decreased fecundity (Preston et al. 2020). Although our study did not investigate such factors, pathogen infections, nutrient imbalances, and genetic changes (eg inbreeding and genetic drift) may have contributed to the observed decline in life history traits. Additionally, the experience of the operators, which was not assessed in this study, could also play a significant role in influencing the output.

In conclusion, despite the constant life history traits observed, the developed protocol allows for the continuous rearing of *H. halys*, although it requires a significant maintenance effort. The protocol demonstrates limitations, particularly affecting the adult stage, where variability in parameters becomes evident starting from F2. A physical constraint, such as the dimension of the rearing chamber may have imposed limits, as we had to restrict the number of rearing cages and Petri dishes per generation. Notably, fecundity, survival, and number of egg masses from F2 onwards are substantially lower than those observed in F0. This decline should be considered, especially for experiments focused on these parameters, such as pesticide testing.

Our study reveals a relatively rapid impact of laboratory rearing on various physiological and morphological traits. When utilizing individuals from ongoing rearing populations for research, it is crucial to consider the effects of inbreeding and its potential impact on fitness, as this factor can significantly influence trial outcomes, resulting in misleading conclusions. A viable solution would be to maintain a parallel, nonisolated subcolony and use individuals from this subcolony for such experiments. This approach would ensure that the results are not compromised by the genetic conditions of individuals derived from a continuous, isolated rearing like the one described in this study.

Our results indicate that the laboratory-reared population stabilized in key parameters, such as fecundity and survival, after a few generations, making it suitable for further research purposes. However, as our study was limited to 8 generations, further testing is needed to confirm this stability. To address issues related to genetic drift, inbreeding or bottleneck events, and to mitigate other factors negatively impacting the mass-reared population of *H. halys*, it is recommended to periodically introduce individuals from wild populations. However, caution is needed, as the introduction of pathogens from wild sources could present additional challenges. Alternatively, starting with a larger initial population could help mitigate these problems. Furthermore, an intrinsic cyclical pattern due to innate seasonality or other factors may exist in *H. halys,* which could be integrated into the standard rearing protocol to enhance outcomes.

Maintaining good “manufacturing practice”, such as ensuring cleanliness of the rearing chambers, rearing boxes, and Petri dishes, and promptly removing dead or harmed individuals, should help stabilize the rearing conditions and improve overall rearing outcomes. Additionally, implementing a quality control system that includes production, process, and product control can further enhance consistency and reliability. Production control monitors output quality (eg egg production and survival), process control standardizes environmental conditions (eg temperature and feeding schedules), and product control ensures that reared individuals meet the necessary standards. These measures will help improve the stability and reproducibility of mass-rearing efforts.
